# Predicting asthma control deterioration in children

**DOI:** 10.1186/s12911-015-0208-9

**Published:** 2015-10-14

**Authors:** Gang Luo, Bryan L. Stone, Bernhard Fassl, Christopher G. Maloney, Per H. Gesteland, Sashidhar R. Yerram, Flory L. Nkoy

**Affiliations:** 1 Department of Biomedical Informatics, University of Utah, Suite 140, 421 Wakara Way, Salt Lake City, UT 84108 USA; 2 Department of Pediatrics, University of Utah, 100 N Mario Capecchi Drive, Salt Lake City, UT 84113 USA

**Keywords:** Asthma control, Predict, Child

## Abstract

**Background:**

Pediatric asthma affects 7.1 million American children incurring an annual total direct healthcare cost around 9.3 billion dollars. Asthma control in children is suboptimal, leading to frequent asthma exacerbations, excess costs, and decreased quality of life. Successful prediction of risk for asthma control deterioration at the individual patient level would enhance self-management and enable early interventions to reduce asthma exacerbations. We developed and tested the first set of models for predicting a child’s asthma control deterioration one week prior to occurrence.

**Methods:**

We previously reported validation of the Asthma Symptom Tracker, a weekly asthma self-monitoring tool. Over a period of two years, we used this tool to collect a total of 2912 weekly assessments of asthma control on 210 children. We combined the asthma control data set with patient attributes and environmental variables to develop machine learning models to predict a child’s asthma control deterioration one week ahead.

**Results:**

Our best model achieved an accuracy of 71.8 %, a sensitivity of 73.8 %, a specificity of 71.4 %, and an area under the receiver operating characteristic curve of 0.757. We also identified potential improvements to our models to stimulate future research on this topic.

**Conclusions:**

Our best model successfully predicted a child’s asthma control level one week ahead. With adequate accuracy, the model could be integrated into electronic asthma self-monitoring systems to provide real-time decision support and personalized early warnings of potential asthma control deteriorations.

## Background

Asthma is the most common pediatric chronic disease [1, 2] and the most frequent reason for preventable pediatric hospitalization [3]. Asthma affects 7.1 million American children [4, 5], accounts for one third of pediatric emergency department (ED) visits [6], and incurs an annual total direct healthcare cost around 9.3 billion dollars [1]. In 2009, 640,000 ED visits, 157,000 hospitalizations, and 185 deaths [4] were due to pediatric asthma. Poor asthma control in children is associated with decreased quality of life [7], increased school absenteeism with work loss for parents [8], and a high hospital readmission rate [9]. Despite its impact, asthma remains a poorly controlled disease [10]. Effective interventions to improve and maintain asthma control are needed.

Asthma control on a patient fluctuates frequently over time due to multiple factors [11–13]. An asthma exacerbation is often preceded by a critical period of decreased asthma control [14]. The critical period often goes unrecognized by patients, caregivers, and physicians [10, 15–18], resulting in missed opportunities for taking preventive interventions such as education and medication prescription and adjustment [15, 17].

Using predictive models can facilitate recognition of impending loss of asthma control before significant symptoms emerge. While many predictive models for diagnosing and treating asthma exist [19], little has been done for predicting asthma control deterioration. Existing models focus on predicting asthma exacerbations, which often represent a late manifestation of persisting loss of asthma control, and have low sensitivities and low positive predictive values [20–24].

In the past, our group developed and validated an asthma control monitoring tool, the Asthma Symptom Tracker (AST) [25]. This self-monitoring tool was designed to assess a child’s asthma control level on a weekly basis. The objective of this study was to develop a model for predicting asthma control deterioration one week ahead, by using scores from previously completed AST assessments [25] in conjunction with patient attributes and environmental variables.

## Methods

### Study setting

The data collected in our AST validation study [25] included demographics and clinical status for patients living primarily in Utah as well as several patients living in Idaho, Nevada, and Wyoming. The patients were recruited during hospitalization for asthma exacerbation. Written informed consent was obtained from each study participant before data were collected on the participant. Environmental exposure data matched by time and location were obtained from multiple regional monitoring stations (federal data sources) [26, 27]. The Germ Watch program [28] of Intermountain Healthcare (Salt Lake City, Utah) provided data for time-matched prevalent viral activity in the Intermountain Region. Intermountain Allergy & Asthma (Salt Lake City, Utah) [29] provided time-matched pollen count and mold level data. Analysis took place at the School of Medicine, University of Utah. The study was reviewed and approved by the Institutional Review Boards of the University of Utah and Intermountain Healthcare.

### Data collection

**Fig. 1:**
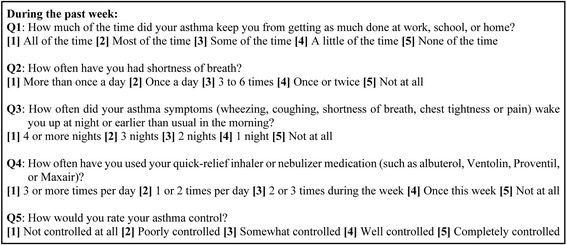
The five questions used in the Asthma Symptom Tracker

As shown in Fig. 1, the AST score is derived from responses to the five questions of a modified Asthma Control Test [18, 25] adapted for weekly assessment of asthma control status. The AST score is the total score of the responses to the five questions, ranges from 5 to 25, and reflects the patient’s asthma control level over the past week. Each patient’s AST assessments were collected for six months. For the current study, a patient was excluded if he/she did not have at least two consecutive AST assessments one week apart. The first AST assessment was completed in the hospital, was almost always categorized as “uncontrolled asthma” reflecting the patient’s status in the pre-hospitalization week, and was excluded from analysis.

Patient demographics included age, race, sex, home address, and health insurance provider. Clinical status included chronic asthma severity level, secondhand smoke exposure, comorbidities, and healthcare use including ED visits and hospital admissions within the six months prior to the index hospital admission. Demographics and clinical status were obtained through extraction from Intermountain Healthcare’s Enterprise Data Warehouse [30] and manual chart review. Median household income and percentage of the population with a college degree based on zip code and health insurance category were obtained as surrogates for socioeconomic status. Low socioeconomic status and Medicaid insurance are known to be associated with poor asthma control in children [31, 32]. Patient home address was used in computing the patient’s environmental exposure via location matching.

Environmental variable data included particulate matter with a diameter of 2.5 micrometers or less (PM_2.5_), PM_10_, carbon monoxide, nitrogen dioxide, sulfur dioxide, ozone, temperature, relative humidity, wind speed, precipitation, dew point, tree pollen count, grass pollen count, weed pollen count, mold level, and activities of each of the following viruses: adenovirus, enterovirus, influenza A virus, influenza B virus, human metapneumovirus, parainfluenza virus types 1, 2, and 3, respiratory syncytial virus, and rhinovirus.

### Data analysis

#### Data preparation

Our primary goal was to predict asthma control deterioration one week ahead. The dependent variable was the patient’s AST score one week following the prediction date, dichotomized to “controlled asthma” or “uncontrolled asthma” based on a cutoff score of >19 = “controlled asthma” [18, 33]. Uncontrolled asthma occurred much less frequently than controlled asthma. This could degrade a predictive model’s performance. To address this issue for the imbalanced dependent variable ([34], Chapter 16), we applied the standard Synthetic Minority Over-sampling TEchnique (SMOTE) [35] to the training set used for estimating a model’s parameters, but not to the test set used for evaluating the model’s performance. Basically, SMOTE over samples the rarer class “uncontrolled asthma” to make the numbers of instances more balanced for the two classes “controlled asthma” and “uncontrolled asthma.” To remove distributional skewedness ([34], Section 3.2), the standard Box-Cox transformation [36] was used to transform each numerical independent variable, which was then normalized by first subtracting its mean and then dividing by its standard deviation ([34], Section 3.2). This makes the data more normal distribution-like.

Evaluation was performed using two approaches. The first approach used standard, stratified 10-fold cross validation ([37], Section 5.3). The data were split into 10 partitions of roughly the same size. In each partition, the proportion of uncontrolled asthma was about the same as that in the full data set. Ten iterations were completed rotating through all partitions, using one for testing and the other nine for training. The 10 performance estimates were averaged to yield an overall performance estimate of the model. In the second approach, the data for each patient’s last AST assessment was used for testing, with the remaining data used as the training set. The performance estimate reflected a model’s performance in making predictions when a patient was in his/her typical clinical asthma status.

#### Performance metrics

As shown in Table 1 and the formulas below, six standard metrics were used to measure a model’s performance: accuracy, sensitivity, specificity, positive predictive value (PPV), negative predictive value (NPV), and Area Under the receiver operating characteristic Curve (AUC). For instance, false negative (FN) is the number of instances of uncontrolled asthma that the model incorrectly identifies as controlled asthma. Sensitivity measures the proportion of all instances of uncontrolled asthma that the model identifies. Specificity measures the proportion of all instances of controlled asthma that the model identifies.

**Table 1:** The error matrix

		Actual level of asthma control	
		Uncontrolled	Controlled
Predicted level of asthma control	uncontrolled	true positive (TP)	false positive (FP)
	controlled	false negative (FN)	true negative (TN)

$$ accuracy=\left(TP+TN\right)/\left(TP+TN+FP+FN\right), $$

$$ sensitivity=TP/\left(TP+FN\right), $$

$$ specificity=TN/\left(TN+FP\right), $$

$$ positive\  predictive\  value=TP/\left(TP+FP\right), $$

$$ negative\  predictive\  value=TN/\left(TN+FN\right). $$

#### Classification algorithms

Our basic predictive model was built using the decision stump classifier, which makes a prediction based on a single independent variable. Advanced predictive models were built using the top six classification algorithms recognized in the machine learning and data mining literature [38, 39]: support vector machine, random forest, multiboost with decision stumps, naive Bayes, *k*-nearest neighbor, and deep learning. Briefly, a support vector machine constructs a hyperplane in a high-dimensional space to separate instances of the two classes. A random forest is an ensemble of decision tree classifiers. Multiboost with decision stumps is an ensemble of decision stump classifiers trained through combining boosting with a variant of bagging. A naive Bayes classifier computes conditional probability by assuming that given the class variable, all independent variables are independent of each other. A *k*-nearest neighbor classifier classifies a new instance based on the classes of the *k* training instances closest to it. An example deep learning classifier is an artificial neural network with multiple hidden layers, i.e., a deep neural network.

Weka [37], the most widely used open-source machine learning and data mining toolkit, was used to build the models. Weka integrates a large set of commonly used machine learning algorithms and methods for handling the imbalanced class problem (i.e., the categories of the dependent variable are imbalanced). For deep learning that is not part of the Weka toolkit, we used the deepnet package in R [40] that implements deep neural network with weights initialized by deep belief network [39, 41, 42].

The classification algorithms and SMOTE require parameter entry. For instance, SMOTE has a parameter controlling the amount of up-sampling [35]. For each predictive model, we chose the parameter values of the corresponding classification algorithm and SMOTE to maximize sensitivity without overly degrading accuracy. Among the six performance measures, sensitivity and accuracy are the primary targets because our main goal is to identify uncontrolled asthma beforehand.

## Results

**Fig. 2:**
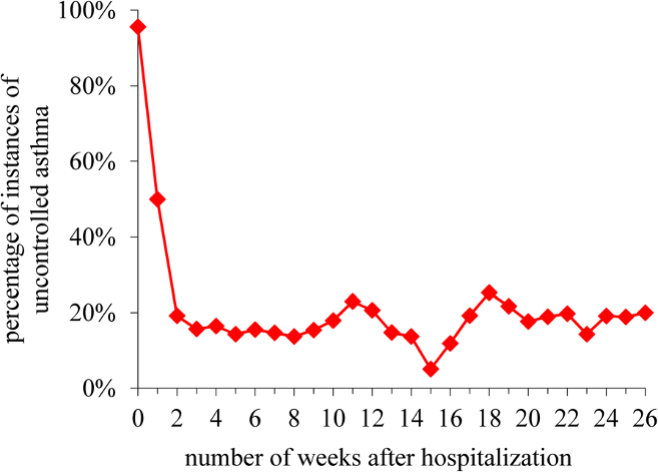
Across all patients, the percentage of instances of uncontrolled asthma over time. Week 0 is the time when the first assessment was obtained on a patient during hospitalization

The original study [25] provided 2912 weekly assessments of asthma control on 210 asthmatic children 2 to 18 years old. After excluding baseline assessments and 30 patients with only one AST assessment, 2617 AST assessments from 180 patients were available for predictive modeling. Table 2 shows patient demographics and baseline clinical characteristics. The percentage of uncontrolled asthma in the AST assessments was 23.5 % overall. As shown in Fig. 2, this percentage was significantly higher in the first week after hospitalization (50 %), then stabilized near 19 % during the remaining follow-up assessments.

**Table 2:** Distribution of the patient attributes

Variable	Category	N
Sex	male	110
	female	70
Age (in years)	2–5	112
	6–10	46
	11–14	19
	15–18	3
Race	Native American	3
	Asian	5
	black	4
	Hispanic	26
	Pacific islander	6
	white	118
	other	14
	unknown	4
State of residence	Idaho	1
	Nevada	4
	Utah	172
	Wyoming	3
Chronic asthma severity level	intermittent	35
	persistent	144
	unknown	1
Insurance category	Medicaid	71
	private	101
	self-paid	8
Exposure to secondhand smoke	yes	35
	no	116
	unknown	29
Presence of any comorbidity	yes	2
	no	178
Previous asthma admission	yes	35
	no	145

Our basic predictive model used the decision stump classifier with one independent variable, the patient’s AST score on the prediction date. As shown in Table 3, the model achieved an accuracy of 73.4-73.9 %, with a low sensitivity of 51.1 % when measured by the method of testing on each patient’s last assessment. Table 3 also lists the performance of the six advanced machine learning classifiers measured by the two evaluation approaches. To improve performance, each of the six advanced classifiers used the five individual component scores of the patient’s AST assessment on the prediction date as independent variables.

**Table 3:** Performance of the different classifiers

Performance of the decision stump classifier						
Evaluation method	Sensitivity	Accuracy	Specificity	AUC	PPV^a^	NPV^a^
10-fold cross validation	67.2 %	73.4 %	74.9 %	0.710	38.1 %	91.0 %
testing on each patient’s last assessment	51.1 %	73.9 %	82.0 %	0.665	50.0 %	82.6 %
Performance of the six advanced classifiers measured by the 10-fold cross validation method						
Classifier	Sensitivity	Accuracy	Specificity	AUC	PPV^a^	NPV^a^
Multiboost with decision stumps	73.8 %	71.8 %	71.4 %	0.761	37.1 %	92.4 %
Support vector machine	71.5 %	72.0 %	72.0 %	0.718	37.0 %	91.8 %
Deep learning	71.6 %	72.3 %	72.5 %	0.744	37.2 %	91.8 %
Naive Bayes	59.8 %	78.1 %	82.3 %	0.777	43.7 %	90.0 %
*k*-nearest neighbor	56.9 %	73.3 %	77.0 %	0.704	36.0 %	88.7 %
Random forest	48.3 %	75.8 %	82.0 %	0.662	37.9 %	87.5 %
Performance of the six advanced classifiers measured by the method of testing on each patient’s last assessment						
Classifier	Sensitivity	Accuracy	Specificity	AUC	PPV^a^	NPV^a^
Multiboost with decision stumps	74.5 %	74.4 %	74.4 %	0.757	50.7 %	89.2 %
Support vector machine	70.2 %	73.3 %	74.4 %	0.723	49.3 %	87.6 %
Deep learning	68.1 %	72.2 %	73.7 %	0.738	47.8 %	86.7 %
Naive Bayes	44.7 %	73.9 %	84.2 %	0.783	50.0 %	81.2 %
*k*-nearest neighbor	48.9 %	73.9 %	82.7 %	0.773	50.0 %	82.1 %
Random forest	38.3 %	75.6 %	88.7 %	0.678	54.5 %	80.3 %

^a^*PPV* positive predictive value; *NPV* negative predictive value

The multiboost with decision stumps classifier had the best performance, with a sensitivity of 73.8 % vs. 74.5 %, an accuracy of 71.8 % vs. 74.4 %, a specificity of 71.4 % vs. 74.4 %, an AUC of 0.761 vs. 0.757, a PPV of 37.1 % vs. 50.7 %, and a NPV of 92.4 % vs. 89.2 %, when measured by the two evaluation approaches, respectively. The support vector machine and deep learning classifiers performed similarly. The naive Bayes, random forest, and *k*-nearest neighbor classifiers performed less well, particularly with respect to sensitivity.

We also used additional independent variables, beyond the component scores of the patient’s AST assessment, collected for this study to improve the advanced predictive models’ performance. These variables included the AST assessment one week prior to the prediction date, the patient attributes, and the environmental variables described above. None of these variables improved the models’ performance (detailed results are not shown). Thus, our best models used only the five individual component scores of the patient’s AST assessment on the prediction date as independent variables, making these models easy to use in practice.

## Discussion

The objective of our study was to develop and test new predictive models for asthma control deterioration using a combination of patient demographic information, clinical information, and environmental variables. Using the multiboost with decision stumps classifier, we were able to successfully predict asthma control deterioration one week ahead with reasonable accuracy, demonstrating the feasibility of predictive modeling. However, performance needs to achieve a higher level of accuracy (e.g., >80 %) and PPV, while maintaining high sensitivity and specificity, before such models can be used to support real-time clinical decision making. If accuracy goals can be met, such a model could be integrated into electronic asthma self-monitoring systems, such as the electronic-Asthma Tracker (e-AT) [43], to provide prediction-based decision support and personalized early warnings of potential asthma control deterioration for asthmatic children. In this case, all independent variables used in the model need to be collected by the electronic asthma self-monitoring system. After the user enters his/her current AST assessment into the system, the system will use the model to predict the user’s asthma control level one week later. If the user is predicted to experience asthma control deterioration, the system will display a personalized warning message to the user.

Although not perfect, our results are encouraging, particularly as the first work on predicting a child’s asthma control deterioration one week ahead. In comparison, despite years of work, existing models on predicting asthma exacerbations have low sensitivities (typically <60 %) and low PPVs (typically <27 %) [20–24], much below those achieved by our best model.

Despite bringing significant burden to patients and the healthcare system, asthma continues to be a poorly controlled disease [10]. Poor asthma control is associated with frequent asthma exacerbations [44]. However, an asthma exacerbation is usually preceded by a critical period of asthma control deterioration [14]. This provides opportunity for interventions if early evidence of deterioration can be identified. Physicians, caregivers, and patients all tend to overestimate the level of asthma control, particularly in children [10, 15–18], resulting in poor recognition of deterioration until an acute exacerbation occurs. One way to identify risk of asthma control deterioration is to develop predictive models. To date, predictive models for deteriorating asthma control have focused on asthma exacerbation, often a late manifestation of loss of asthma control [44].

Using environmental variables, patient attributes, and the patient’s daily peak expiratory flow rate (PEFR) in the previous few days, Lee *et al*. [45] built a model to predict an asthma exacerbation. Lee’s model, however, did not predict asthma control deteriorations preceding an exacerbation, and thus cannot be used to support early intervention to prevent clinical deterioration. In addition, although monitoring PEFR is commonly used to identify early signs of asthma control deterioration, it has several limitations including: (1) the measurement is labor intensive, impacting compliance [43]; (2) PEFR is effort dependent [46] with low reproducibility [47–49]; (3) PEFR primarily assesses large airway airflow and underestimates airflow limitations in medium and small airways [49, 50]; (4) PEFR goals are usually based on the patient’s best PEFR [49], which may differ from the predicted or desired goals [43]; and (5) PEFR goals increase with age and height [51] and must be re-calculated periodically, which is often overlooked [43].

In our AST, asthma control assessments are based on the Asthma Control Test questionnaire adapted for weekly assessment of asthma control status. Thus, we avoid using the more difficult and less accessible forced expiratory volume in 1 second (FEV), forced vital capacity (FVC), and PEFR, and their limitations for use in children.

In our study, the multiboost with decision stumps, support vector machine, and deep learning classifiers performed similarly and achieved reasonable accuracy, sensitivity, specificity, AUC, and NPV. All of these three classifiers could predict a child’s asthma control deterioration one week ahead with reasonable accuracy. The naive Bayes, random forest, and *k*-nearest neighbor classifiers performed less well, particularly with respect to sensitivity.

The AST assessment reflects the patient’s asthma control level over the past week. Successive patient AST assessments are highly correlated with each other. Also, adding the AST assessment one week prior to the prediction date does not improve the models’ performance. We would expect that obtaining AST assessments on a patient more than once per week will not increase prediction accuracy, as information contained in additional AST assessments has already been included in the AST assessments on the prediction date and one week prior to the prediction date.

Our study has several limitations. First, the patients were recruited during hospitalization for asthma exacerbation. Each year, only ~1.6 % of asthmatic children are hospitalized [4]. As is typical with predictive modeling, our models’ performance is affected by the percentage of uncontrolled asthma in AST assessments. The percentage may be lower in patients not hospitalized than in patients hospitalized. A model’s performance usually degrades as the percentage of uncontrolled asthma in AST assessments decreases. It remains to be seen how our models will perform on patients not hospitalized. Second, we had a small sample size and were limited by the number of patient attributes and environmental variables. Collecting additional AST assessments and patient attributes can potentially improve the models’ performance. Such attributes might include information on allergies, parental asthma [20], healthcare access, the number of prescribing providers [21], viral infection severity [52], compliance with asthma controller medications, and other known predictors of asthma control such as pet exposure [53]. Third, our sample is relatively homogenous. For instance, 66 % of the patients are white. The small sample size limits our capability to (a) detect the association between a variable that is relatively homogenous and the asthma control level, and (b) conduct subgroup analysis to determine whether prediction accuracy differs among various patient subgroups (e.g., by race or by chronic asthma severity level). Fourth, our environmental variable data came from regional monitoring stations and may not reflect a patient’s actual exposures [45, 54, 55]. Accurate measurement of environmental exposures would benefit from using a personal exposure monitor [46, 55–57] and may help increase the models’ performance. We did find environmental variables correlated with an asthmatic child’s level of asthma control, but the correlation was relatively weak [58, 59]. By including the environmental variables, but not the patient’s AST score, in the predictive models, our best model achieved a low sensitivity of 41.7 % and a low AUC of 0.593.

To better understand our predictive models’ performance, we used two evaluation methods simultaneously. These two methods address different situations. When a choice among multiple predictive models needs to be made, these two evaluation methods can provide insights into which model is most suitable for the clinical situation and desired outcome. The patient’s AST score on the prediction date reflects the patient’s asthma control level, and hence can be an approximate surrogate for the environmental variables in the previous seven days assuming they have a non-trivial influence on asthma control. We felt that this, and the high correlation between successive patient AST assessments, rendered our modeling attempts less successful than desired. It is likely that AST assessments have an overpowering influence on the prediction in comparison to environmental variables and other patient attributes, making their relative contributions insignificant. This effect was compounded by our small sample size.

As with any intervention relying on patient-reported data, our technology’s utility hinges on patient adherence to continuous reporting of their data. If patients cannot obtain benefits from efforts expended on reporting their data, adherence is likely to wane. The functionality of predicting asthma control deterioration, once done accurately and incorporated into the e-AT, will provide direct benefits to patients and may help improve patient adherence.

We have several goals in mind for future work. First, we would like to improve the models’ performance. This will be accomplished by: (1) increasing the sample size to improve the capability to detect the association between a variable and the asthma control level, (2) obtaining additional patient attributes among the known predictors of asthma control, (3) collecting additional environmental variables, such as pet exposure at the patient’s home, (4) collecting patient-specific environmental variables with portable monitors rather than estimating from regional monitoring stations, and (5) integrating patient pharmacogenomics information relating to medication metabolism.

Second, we would like to investigate how our models will perform in the ambulatory setting with non-hospitalized patients.

Third, we would like to probe the possibility of making an earlier prediction. In general, the earlier and the more accurate the prediction, the more useful the prediction will be for clinical decision making.

Fourth, we would like to extend our predictive models to incorporate intervention information, in a way similar to that in interrupted time series models or intervention models [60]. Our current models consider no intervention information. However, once prediction-based warnings start to be provided to an asthmatic child, the child may be given a preventive intervention. The intervention will impact the child’s asthma control level in the future and thus needs to be considered in the predictive model.

## Conclusions

Our best models predicted with reasonable accuracy a child’s asthma control level one week ahead. With improvements in accuracy, the models can be integrated into electronic asthma self-monitoring systems to provide real-time decision support and personalized early warnings on potential asthma control deterioration for asthmatic children. This will allow implementing preventive actions to reduce asthma exacerbations, improve clinical outcomes, increase quality of life, and reduce healthcare cost.

## Ethics approval

The study was reviewed and approved by the Institutional Review Boards of the University of Utah and Intermountain Healthcare.

## Abbreviations

AST: Asthma symptom tracker
AUC: Area under the receiver operating characteristic curve
e-AT: Electronic-asthma tracker
ED: Emergency department
FN: False negative
FP: False positive
NPV: Negative predictive value
PEFR: Peak expiratory flow rate
PPV: Positive predictive value
SMOTE: Synthetic minority over-sampling technique
TN: True negative
TP: True positive
